# Dutch sensory modality norms

**DOI:** 10.3758/s13428-021-01656-9

**Published:** 2021-09-10

**Authors:** Laura J. Speed, Marc Brybaert

**Affiliations:** 1 Centre for Language Studies, Radboud University, Nijmegen, Netherlands; 2 Department of Experimental Psychology, Ghent University, Ghent, Belgium

**Keywords:** Sensory norms, Mental simulation, Concepts, Perceptual modalities

## Abstract

Many words are strongly connected to the senses, such as vision, taste, and touch. In order to facilitate research on language and the senses, large sets of linguistic stimuli and their corresponding measures of sensory associations should be available. To aid in such investigations, we present a new set of sensory modality norms for over 24,000 Dutch words. The sensory norms comprise perceptual strength ratings in six perceptual modalities: audition, gustation, haptics, olfaction, vision, and interoception. The new norms improve on existing Dutch sensory norms in three ways: 1) they significantly expand on the number of words rated; 2) they include multiple word classes; 3) they add a new perceptual modality: interoception. We show that the sensory norms are able to predict word processing behavior and outperform existing ratings of sensory experience: concreteness and imageability. The data are available via the Open Science Framework (https://osf.io/ubvy2) and serve as a valuable resource for research into the relationship between language and perception.

**Supplementary Information:**

The online version contains supplementary material available at 10.3758/s13428-021-01656-9.

Sensory information extracted from experience critically underlies our understanding of the world. Theories of embodiment propose that sensory information contributes to language understanding as well (e.g., Barsalou, 1999; Meteyard et al., 2012; Vincent-Lamarre et al., 2016). This is supported by numerous behavioral and neuroimaging studies finding that sensory cortices are activated during word comprehension (e.g., Martin et al., 1995; Meteyard et al., 2008; Pulvermuller & Hauk, 2005; Zwaan et al., 2002), although some findings have proved to be difficult to replicate (e.g., Kaschak et al., 2018).

Most evidence for a role of sensory information in language comprehension comes from visual and auditory modalities, leaving open the question how relevant the other senses (i.e., gustation, haptics, olfaction) are to word meaning (Speed & Majid, 2018, 2019). This highlights the importance of assessing the contribution of individual sensory modalities to meaning, and making comparisons across the senses. One way to measure the role of the senses in word meaning is to ask participants to rate words on their perceptual associations across the sensory modalities. This provides a fine-grained, continuous measure of words’ sensory make-up, which can be used to make fine-grained assessments in experiments.

Human ratings of words’ perceptual associations are of growing importance, for the design and analysis of experiments and to learn more about words’ perceptual underpinnings. In recent years, several databases of sensory modality norms have been released for a number of languages (Chen et al., 2019; Filipović Đurđević et al., 2016; Lynott et al., 2020; Lynott & Connell, 2009, 2013; Miklashevsky, 2018; Morucci et al., 2019; Speed & Majid, 2017; van Dantzig et al., 2011; Vergallito et al., 2020; Winter, 2016). Sensory modality norms reflect participant judgments of how concepts are related to the sensory modalities. For each modality, participants are asked to rate to what extent the meaning of a word is associated with perceptual experience in that modality. Words can then be categorized into a dominant modality (i.e., visual, auditory, gustatory, olfactory, haptic, interoceptive) based on the highest modality strength. For example, *purple* is a word strongly associated with visual experience and *citrusy* is a word strongly associated with gustatory experience (Lynott & Connell, 2013). Each word is also associated with a vector of perceptual strength across modalities, as well as a modality exclusivity score, which indicates how multimodal the concept is.

Sensory ratings can be used empirically in at least three important ways: 1) in linguistic analyses of the mental lexicon, testing the role of the sensory modalities in word meanings (e.g., Strik Lievers & Winter, 2018; Winter, 2016, 2019; Winter et al., 2018); 2) as predictors in large mega-studies of word processing (Connell & Lynott, 2012, 2014); 3) to select stimuli in psycholinguistic experiments (e.g., Speed & Majid, 2018). In this way, ratings can increase reliability of experimental data (Balota et al., 2004) and allow an assessment of the simultaneous impact of perceptual information across the senses on lexical processing.

In general, behavioral patterns observed using sensory modality ratings of words reflect patterns seen in human perception, thereby supporting the validity of the ratings. In particular, sensory modality ratings have been showed to be significant predictors of behavioral responses to words across experiments. For example, Lynott and Connell (2009) evaluated sensory modality ratings of English object property words using the modality switch cost experiment (based on Pecher et al., 2003). Replicating the original finding of Pecher et al. (2003), participants took longer making property verifications when switching from one modality to another (e.g., visual *apple* – *shiny* to auditory *leaves* – *rustling*), compared to making verifications within the same modality. Critically, since the items were selected to be highly unimodal based on the modality ratings (i.e., predominantly associated with only one perceptual modality), the effect size was substantially larger than in the original study. Connell and Lynott (2014) used an expanded set of the same norms to investigate the effect of perceptual attention on conceptual processing. They found that words rated highly on visual strength (e.g., *cloudy*) were facilitated in a lexical decision task and reading aloud task, tasks that direct attention towards vision. Similarly, words rated highly on auditory strength (e.g., *noisy*) were facilitated in the reading aloud task, because this task also directs attention to the auditory modality. Another study using a lexical decision task found that processing odor-related words may differ to processing words strongly related to other modalities. Speed and Majid (2017) used sensory modality ratings of Dutch nouns to investigate the putative mapping of the senses onto near and far space, with taste, smell and touch typically associated with near, or proximal, space, and sight and sound typically associated with far, or distal, space. Words were presented onscreen in a “near” position (large font, low on screen) or a “far” position (small font, high on screen). Responses for words dominant in vision and audition (distal words) were slower when presented in the near position compared to the far position. Conversely, response times to words dominant in olfaction were facilitated in the near position compared to the far position. This suggests that spatial experience associated with individual perceptual modalities is activated during semantic processing. Sensory modality ratings have also been shown to predict surface lexical characteristics of words: word length, word-form distinctiveness, and word frequency (Lynott & Connell, 2013). For example, English words strongly associated with auditory experience tend to be longer, and words related to haptic experience tend to be shorter.

Here we expand on existing sensory modality ratings by providing new ratings for a large set of Dutch words. We build on the existing sensory modality norms for Dutch words (Speed & Majid, 2017) in several respects. Firstly, the new dataset contains significantly more words (24, 036 compared to 485). Secondly, a wider array of word class is covered here. Finally, the new dataset contains ratings on the modality of interoception, which were not collected in Speed and Majid (2017). Interoception is the perceptual modality related to sensations inside the body, including sensations from the heart, lungs, and stomach (Connell et al., 2018). It is thought to be more important for abstract than concrete concepts, and particularly important for emotional concepts (Connell et al., 2018).

Following previous work (Connell & Lynott, 2012; Miklashevsky, 2018; Vergallito et al., 2020) we also set out to compare the new sensory norms to existing ratings of concreteness and imageability. Concreteness and imageability have traditionally been found to facilitate lexical processing, i.e., the so-called “concreteness effect” (e.g., James, 1975; Kroll & Merves, 1986). Concrete words, thought to be strongly connected to a wealth of sensory information, should be processed faster than abstract concepts (but see e.g. Brysbaert et al., 2016), that are instead associated more with verbal information (Paivio, 1986), or weakly connected to a wide range of different contexts (Schwanenflugel et al., 1988). However, criticisms of concreteness and imageability ratings have been raised, suggesting that participants’ ratings on these scales may be biased. Imageability ratings, for example, tend to be visually biased at the expense of the other perceptual modalities (Connell & Lynott, 2012). We therefore compare the sensory norms with ratings of concreteness and imageability. If concreteness and imageability ratings accurately reflect sensory experience across the perceptual modalities, then sensory ratings across all modalities should significantly predict ratings of concreteness and imageability. In contrast, if ratings of concreteness and imageability are biased towards the visual modality, then ratings for modalities other than vision should not predict (or negatively predict) ratings of concreteness and imageability. Connell and Lynott (2012) found that concreteness and imageability ratings of English words do not reflect sensory experience across all modalities. Both concreteness and imageability ratings were positively predicted by ratings in the olfactory, visual, and haptic modality, but negatively predicted by auditory and gustatory ratings.

We also compare concreteness and imageability ratings to the sensory norms in their ability to predict lexical processing. If the often-observed concreteness effect (e.g., James, 1975; Kroll & Merves, 1986) is explained by perceptual information underlying concepts, then sensory ratings should also predict lexical processing. If sensory ratings better capture the perceptual information underlying concepts, then sensory ratings should be a better predictor of lexical processing than ratings of imageability and concreteness. Connell and Lynott (2012) found that sensory ratings (specifically maximum perceptual strength) outperformed both concreteness and imageability in predicting lexical decision response time and accuracy.

Finally, we take advantage of having ratings across word classes, and aim to assess the differential contribution of perceptual information to adjective, noun, and verb concepts. Lynott and Connell (2013) propose that noun concepts are typically more multimodal than adjective concepts because they are associated with multiple properties. For example, a *blanket* can be *warm*, *blue*, *itchy*, and *smelly*, and therefore is strongly associated with haptics, vision, and olfaction (Lynott & Connell, 2013). In contrast, adjectives tend to be related to only a single perceptual modality. Winter (2019) proposes that adjectives have less sensory content than nouns, and are instead associated more with evaluative content, reflecting a trade-off between perceptual and evaluative content in word meaning. The sensory ratings of English nouns and adjectives confirm these views, with nouns having an average modality exclusivity of 39.2% (Lynott & Connell, 2013) and adjectives a higher average modality exclusivity of 46.% (Lynott & Connell, 2009).

In what follows, we begin by presenting the new sensory norms and assessing the patterns of association across modalities. As a check of the reliability of the new norms, we compare the new dataset with ratings for overlapping words in Speed and Majid (2017). We then compare the sensory ratings with ratings of concreteness and imageability. Finally, we compare the sensory ratings’ predictability of lexical decision accuracy and response time.

## Study 1: Sensory norming

### Method

#### Materials

The materials consisted of 24,036 words that were rated on word concreteness and age of acquisition (AoA) in Brysbaert et al. (2014), and that were known to 90% of the raters at that time. They were randomly distributed over 24 lists of around 1000 words.

#### Participants

Participants were students from Ghent University (18–26 years old; two-thirds female). They were members of the participant pool or they contacted us after word of mouth. Each participant completed a list of 1000 words on all six ratings. This took on average 3.5 h for which participants were paid €40. Participants with ratings that correlated with those of the rest were given the opportunity to complete up to five lists under the same conditions. A new list was given when the previous one was returned and validated. All returned lists were valid, although several participants did not return their list (arguably because they stopped after a few lines). Participants had to be native speakers of Dutch.

#### Procedure

Participants could complete the list at their own pace at home. They were asked to find a quiet place and told they could complete the list in as many sessions as suited them. They received an Excel file with 1000 words and were asked to what extent they experienced the entity referred to by the word with their various senses (“In welke mate ervaar je …” [To what extent do you experience…]). For each sense, they were asked to use a number from 0 (helemaal niet van toepassing [not at all]) to 5 (heel erg van toepassing [very much]). Next to the words, there were seven columns entitled:

- door aanraking (by touching)
- door horen (by hearing)
- door zien (by seeing)
- door ruiken (by smelling)
- door proeven (by tasting)
- door sensaties binnen in het lichaam (by sensations inside the body)
- onbekend woord (unknown word)

The last column was added to make sure that no ratings would be given for words unknown to the rater. We also alerted the raters to the fact that it was possible they would have to use a lot of small digits because the word did not refer to anything that could be sensed, and that this was not a problem, as long as they took the task seriously. We felt that otherwise, raters might be inclined to give higher numbers than they felt.

Each of the 24 lists was given to ten raters. At that moment, the intraclass correlation was calculated. If the correlation was lower than .8, extra raters were contacted. In total, 269 lists had to be completed, with an average of 11.25 participants per list. The maximum number of participants per list was 13 and the minimum was 10.

### Results

The data discussed in this article and the analyses conducted are freely available on the Open Science Framework website (https://osf.io/ubvy2). Summary statistics over all words for each dimension can be found in Table 1. As observed repeatedly across sensorimotor norms (e.g., Chen et al., 2019; Filipović Đurđević et al., 2016; Lynott et al., 2020; Lynott & Connell, 2009, 2013; Miklashevsky, 2018; Morucci et al., 2019; Speed & Majid, 2017; Vergallito et al., 2020) words were rated as primarily experienced in the visual modality, and experienced least in the gustatory modality. Table 2 displays mean ratings per dimension for words dominant in each modality. The greatest number of words were dominated by vision, and the smallest number by olfaction. In addition, 583 words were equally dominant in more than one modality. Table 3 displays a sample of nouns and their mean ratings across modalities.

**Table 1:** Summary statistics of ratings per dimension

Modality	M	SD	SE
Auditory	1.13	0.99	0.006
Gustatory	0.25	0.77	0.005
Haptic	1.02	1	0.006
Olfactory	0.31	0.68	0.004
Visual	2.64	1.06	0.007
Interoceptive	0.76	0.84	0.005

**Table 2:** Distribution of words over the six dominant modalities, with mean ratings and modality exclusivity scores. Rows refer to ratings in each sensory modality, and columns refer to words’ dominant modality

Variable	Dominant modality					
	Auditory	Gustatory	Haptic	Olfactory	Visual	Interoceptive
Auditory rating	2.79	0.25	0.77	0.46	0.92	1.17
Gustatory rating	0.05	4.07	0.20	1.06	0.15	0.13
Haptic rating	0.41	1.70	3.26	1.01	1.07	0.49
Olfactory rating	0.07	2.43	0.25	3.59	0.26	0.13
Visual rating	1.62	3.11	2.61	2.20	2.92	1.65
Interoceptive rating	0.80	0.45	0.91	0.68	0.54	2.52
Modality exclusivity	50.0%	33.5%	41.1%	41.0%	52.0%	42.2%
*N*	2567	613	589	161	17498	2026

**Table 3:** Sample of words with profile of perceptual strength ratings across dimensions (0–5) and modality exclusivity score

	**Auditory**	**Gustatory**	**Haptic**	**Olfactory**	**Visual**	**Interoceptive**	**PoS**	**Modality**	**Modality exclusivity**
*stopteken*	0.27	0.00	0.09	0.00	4.64	0.45	Noun	Visual	85%
stop sign									
*geparfumeerd*	0.10	0.30	0.00	5.00	0.10	0.50	Adjective	Olfactory	83%
perfumed									
*meekijken*	0.31	0.00	0.15	0.00	4.00	0.31	Verb	Visual	84%
watch									
*gekibbel*	3.62	0.08	0.15	0.08	2.23	0.92	Noun	Auditory	50%
bickering									
*zoet*	0.00	4.18	0.27	2.18	0.55	1.18	Adjective	Gustatory	50%
sweet									
*deprimeren*	1.73	0.00	0.09	0.00	1.55	3.55	Verb	Interoceptive	51%
depress									
*kots*	1.83	3.17	1.92	4.50	3.92	2.42	Noun	Olfactory	15%
puke									
*verfrissend*	0.54	1.77	1.92	1.85	1.38	1.31	Adjective	Haptic	16%
refreshing									
*opwarmen*	0.82	1.09	2.00	0.82	1.64	1.91	Verb	Haptic	14%
warm up									

Table 2 also includes mean modality exclusivity for words dominant in each modality. Modality exclusivity reflects the degree to which a concept is associated with a single perceptual modality. It is calculated by dividing the range of ratings for each item, by the sum of ratings for each item. Modality exclusivity scores range from 0%, indicating a fully multimodal concept, to 100%, indicating a unimodal concept. In the present ratings, *zintuigen* (*senses*) had the lowest modality exclusivity score of 2.5%. Forty-seven words had a modality score of 100%, all of which were exclusively associated with vision (e.g., *kolom* (*column*), *glimp* (*glimpse*), *zestiende* (*sixteenth*)). One word, *mettertijd* (*over time*) had no modality exclusivity score because it had a mean rating of 0 across all dimensions. We subsequently removed this word from further analyses. Words dominant in vision were the most unimodal (52%), followed by audition (50%), interoception (42.2%), haptics (41.1%), and olfaction (41.0%), with words dominant in gustation (33.5%) the most multimodal. Overall, words had a mean modality exclusivity score of 49.7% (SD = 14.3%). This value is slightly higher than those observed in previous sensorimotor norms across multiple words classes (43.5%: Lynott et al., 2020; 40.6%: Vergallito et al., 2020) as well as specifically nouns (39.2%: Lynott & Connell, 2013; 47%: Speed & Majid, 2017) and adjectives (46.1%: Lynott & Connell, 2009; 46.4%: Chen et al., 2019). Looking separately by word class, here number words had the highest modality exclusivity: 64.4% (SD = 12.2%, *N* = 59), followed by function words: 52.1% (SD = 16.3%, *N* = 617), nouns: 50.9% (SD = 14.3%, *N* =14397), verbs: 47.6% (SD = 12.9%, *N* = 4747), and adjectives were rated the most multimodal: 47.5% (SD = 14.6%, *N* = 4217). This contrasts with the proposal that nouns are more multimodal than adjectives (Lynott & Connell, 2013; Winter, 2019). Note however that Winter (2019) compared modality exclusivity against a chance baseline derived using a permutation-based approach, which could possibly explain any observed differences.

Correlation analyses were conducted between ratings in each modality (see Supplementary Material). All modalities were significantly correlated with each other. Positive correlations were observed between visual and haptic, gustatory, and olfactory ratings, between haptic and gustatory and olfactory ratings, between gustatory and olfactory ratings, and between auditory and interoceptive ratings. Negative correlations were observed between auditory ratings and visual, haptic, gustatory and olfactory ratings, and between interoceptive ratings and visual, haptic, gustatory, and olfactory ratings. As observed previously across languages (Chen et al., 2019; Lynott et al., 2020; Lynott & Connell, 2009, 2013; Miklashevsky, 2018; Speed & Majid, 2017; Vergallito et al., 2020), olfactory and gustatory ratings had the strongest positive correlation, reflecting their joint involvement in flavor. In Lynott et al. (2020), interoceptive ratings were negatively correlated with visual and haptic ratings, whereas here interoceptive ratings were negatively correlated with ratings in all modalities except audition. This negative correlation may reflect the fact that interoceptive experiences occur inside the body and are therefore not associated with the external senses. The positive association between interoceptive ratings and auditory ratings however may be because many words dominant in interoception include abstract concepts such as *geweten* (*conscience*), *intuïtie* (*intuition*)*, overdenken* (*think over*), which are likely to be associated with an inner voice (Borghi et al., 2018; Mazzuca & Borghi, 2019), and therefore audition. The patterns of correlations are similar when looking separately by word class (see Supplementary Material), except that for adjectives, interoceptive ratings are only significantly negatively correlated with visual ratings and positively correlated with auditory ratings, and for verbs, the negative correlation between auditory ratings and gustatory and olfactory ratings is not significant.

#### Comparison with Speed and Majid (2017)

To assess the reliability of the Dutch sensory modality norms, we compared our norms with those of Speed and Majid (2017). Four hundred and two of the words in the present set also had ratings in Speed and Majid (2017). We calculated correlations between ratings in each dimension (see Table 4; note that Speed and Majid (2017) did not collect ratings of interoception). Our ratings were highly correlated with Speed and Majid (2017) across all five dimensions (see Table 4). However, the correlation between visual ratings was the weakest. Words were given lower visual ratings in the present data set than in Speed and Majid (2017). This could be due to differences in the overall sample of stimuli. Speed and Majid (2017) presented participants with nouns specifically selected to be highly associated with the five sensory modalities, whereas the present stimuli include a range of word classes with no aim in relation to words’ perceptual associations. In Lynott and Connell (2009), where adjectives were also purposefully selected to cover a range of perceptual experience, average ratings of visual strength are also higher (3.57 vs. 2.64). We also calculated similarity between the datasets in terms of cosine similarity, following Winter (2019). Cosine similarity reflects the similarity between vectors of ratings across sensory modalities, rather that only pairwise comparisons within a modality. We calculated cosine similarity for each word and then calculated the average across words. The average cosine similarity for words in Speed and Majid (2017) and the current data was 0.98. Therefore ratings are highly similar across datasets.

**Table 4:** Correlations between new ratings and Speed and Majid (2017) for each modality

Modality	*r*
Auditory	.933
Gustatory	.973
Olfactory	.907
Haptic	.739
Visual	.582
Exclusivity	.843

all *p*s < .001

We also compared words’ dominant modalities across the two datasets. Seventy words from Speed and Majid (2017) changed dominant modality (17%; see Table 5). The most common change was to vision as the dominant modality, which is in line with a greater proportion of words dominant in vision in this data set compared to Speed and Majid (2017).

**Table 5:** Changes from dominant modality in Speed and Majid (2017)

Modality Speed and Majid (2017)	No change	Changed	New modality					
			Auditory	Gustatory	Olfactory	Haptics	Vision	Joint
Auditory	29	9	-	-	-	1	6	2
Gustatory	101	21	-	-	2	-	12	7
Olfactory	22	13	-	3	-	-	9	1
Haptics	25	15	-	-	-	-	15	-
Vision	223	13	5	-	-	7	-	1
Joint	2	2	-	1	-	-	1	-

## Study 2

Following Connell and Lynott (2012), we assessed to what extent modality-specific ratings in the six perceptual modalities overlap with ratings of concreteness and imageability. Although ratings of concreteness and imageability are strongly correlated here (*r* = .76) they are thought to reflect somewhat different aspects of concepts.

### Study 2a: Comparing modality ratings with concreteness ratings

Following Connell and Lynott (2012), we assessed to what extent modality-specific ratings in the six perceptual modalities overlap with ratings of concreteness. As described by Connell and Lynott (2012), concreteness ratings do not always reliably predict semantic ratings, despite their widespread use in psycholinguistics. One reason for this may be that when asked to rate the concreteness of words, participants fail to adequately consider all perceptual modalities.

### Data and analysis

We used the concreteness ratings collected by Brysbaert et al. (2014), which were available for all words in our sample. We conducted stepwise regression with tenfold cross-validation in R (R Core Team, 2013) with the caret package (Kuhn, 2009). Concreteness was the dependent variable and ratings of auditory, gustatory, haptic, olfactory, visual and interoceptive strength were predictors. Following Connell and Lynott (2012), we also split the data into concrete (concreteness rating 3–5, *N* = 11,695) and abstract (concreteness rating < 3, *N* = 12,343) words and ran separate regression models on these data. The same analysis was conducted by word class and can be found in the Supplementary Materials.

### Results and discussion

The best fitting model contained all six modalities, *F*(6, 24029) = 6550, *p* < .001, *R*^2^ = .628, RMSE = .629. Visual, auditory, haptic, gustatory and olfactory ratings were significant positive predictors of concreteness, whereas interoceptive ratings negatively predicted ratings of concreteness. Standardized regression coefficients are displayed in Table 6. The regression coefficients suggest that ratings of concreteness are most strongly described by ratings of visual and haptic experience, and less so by the other perceptual modalities. This supports the proposal that concreteness ratings are biased towards specific perceptual modalities (Connell & Lynott, 2012).

**Table 6:** Standardized regression coefficients (weights) for each dimension as predictors of concreteness. All coefficients significant at *p* < .05

**Rating**	**Visual**	**Auditory**	**Haptic**	**Gustatory**	**Olfactory**	**Interoceptive**	***R***^**2**^
Concreteness	0.442	0.049	0.314	0.048	0.128	– 0.196	.620
Concreteness (Concrete)	0.297	0.031	0.320	0.089	0.169	– 0.134	.375
Concreteness (Abstract)	0.361	0.073	0.189	0.025	-	– 0.189	.245

For concrete words, perceptual strength in all six modalities significantly contributed to the model of concreteness ratings *F*(6, 11687) = 1175, *p* < .001, *R*^2^ = .376, RMSE = .461. Again, visual, auditory, haptic, gustatory and olfactory ratings were significant positive predictors of concreteness, whereas interoceptive ratings negatively predicted ratings of concreteness (see Table 6). Visual and haptic ratings were again the strongest positive predictors, with olfaction, audition, and gustation neglected in comparison.

For abstract words, perceptual strength in five modalities contributed to the regression model of concreteness ratings *F*(6, 12336) = 800, *p* < .001, *R*^2^ = .245, RMSE = .390, with ratings of olfaction excluded. The remaining dimensions were significant positive predictors of concreteness, except for interoceptive strength which was negative (see Table 6). In other words, stronger ratings on interoception relate to words rated as more abstract. Visual and haptic ratings were again the strongest positive predictors. In comparison with Connell and Lynott (2012), the relationship between ratings of modality strength and concreteness appears more stable, with only ratings of olfaction behaving differently across abstract and concrete concepts. Interestingly, interoception, which is also negatively correlated with visual, haptic, olfactory, and gustatory ratings, was the only perceptual modality to have a negative association with concreteness. Words more strongly associated with sensations inside the body tend to be more abstract.

### Study 2b: Comparing modality ratings with imageability ratings

We next assessed to what extent modality-specific ratings in the six perceptual modalities overlap with ratings of imageability. Although imageability ratings should reflect the ability to mentally image across sensory modalities the ratings tend to be visually biased (Connell & Lynott, 2012).

#### Data and analysis

Imageability ratings were retrieved from De Deyne and Storms (2008), Van Loon-Vervoorn (1985), and Verheyen et al. (2020). No imageability ratings were available for function words. Where there was overlap in the three databases, we chose the most recent rating of imageability. In total, there were imageability ratings available for 5658 of the words in our dataset. We again conducted stepwise regression with tenfold cross-validation in R (R Core Team, 2013) with the caret package (Kuhn, 2009). Imageability was the dependent variable and ratings of auditory, gustatory, haptic, olfactory, visual and interoceptive strength were predictors. The same analysis was conducted by word class and can be found in the Supplementary Materials.

#### Results and discussion

Perceptual strength on all six modalities significantly contributed to the model, *F*(6, 5651) = 869.7, *p* < .001, *R*^2^ = .480, RMSE = .90. Visual, auditory, haptic, gustatory, and olfactory ratings were significant positive predictors of imageability, whereas interoceptive ratings negatively predicted ratings of imageability. Standardized regression coefficients are displayed in Table 7. Visual ratings were by far the strongest predictor, confirming the suggestion that whilst the measure of imageability was intended to cover imagery across all modalities, instead ratings tend to favor visual imagery (Connell & Lynott, 2012).

**Table 7:** Standardized regression coefficients for each dimension as predictors of imageability. * = significant at *p* < .001

**Rating**	**Visual**	**Auditory**	**Haptic**	**Gustatory**	**Olfactory**	**Interoceptive**	***R***^**2**^
Imageability	0.50*	0.06*	0.22*	0.01*	0.10*	– 0.06*	.479
Imageability: (High)	0.41*	0.04*	0.20*	0.00	0.12*	– 0.08*	.340
Imageability: (Low)	0.16*	0.08*	0.07*	-	0.07*	-	.053

We then split the data into high and low imageability (high rating 4 –7, *N* = 4440, low rating < 4, *N* = 1218) and ran separate regression models on these data. For high imageability, the best-fitting model included all six predictors, *F*(6, 4433) = 381.4, *p* < .001, *R*^2^ = .340, RMSE = .656. Ratings in all modalities were significant positive predictors, except interoception which was negative, and gustatory which was not significant. Observing the coefficients, again visual ratings were the strongest predictor. For low imageability, gustatory and interoceptive ratings were excluded from the model. The remaining four modalities significantly contributed to the model of imageability, *F*(4, 1213) = 17.01, *p* < .001, *R*^2^ = .053, RMSE = .531. All modalities were positive predictors of imageability, with visual ratings again contributing the most (see Table 7).

## Study 3a: Does perceptual strength outperform concreteness ratings in predicting word processing performance?

As in Connell and Lynott (2012), we compared the performance of sensory ratings and ratings of concreteness in predicting word processing.

### Data and analysis

We used lexical decision raw response time and accuracy taken from the Dutch Lexicon Project 2 (Brysbaert et al., 2016) as a measure of word processing, and conducted hierarchical regression to assess the unique variance in lexical decision performance explained by ratings of perceptual strength and concreteness. For each hierarchical regression, a baseline model included the following variables taken from Brysbaert et al. (2016): word frequency, word prevalence, number of letters, number of syllables, number of phonemes, OLD20 (similarity to other words), and position of speech. We then tested models of the independent effect of each variable, by adding each variable to a model containing the other^1^. We also tested the independent effects of average perceptual strength, modality exclusivity, Minkowski3 (sensory strength across modalities with the effect of weaker modalities attenuated), as well as entering ratings of the six individual modalities in a single block, to assess which is the best perceptual model of word processing. Connell and Lynott (2012) found that maximum perceptual strength was the best predictor of lexical decision and naming performance (compared to other compressed measures of sensory ratings), and Lynott et al., (2020) found Minkowski3 was the best predictor. For each model we estimated RMSE using tenfold cross-validation in R (R Core Team, 2013) with the caret package (Kuhn, 2009). To check for overfitting, we trained each model on 80% of the data and tested it on 20%. We also report RMSE for the test set (RMSE_test_).

### Results and discussion

When analyzing independent effects on response time, all variables were significant. Concreteness was the strongest unique predictor of response time, however the regression coefficient was positive, which is a reversal of the typical concreteness effect. In contrast, measures of maximum perceptual strength, average perceptual strength, and Minkowski3 had negative regression coefficients: the more strongly a word is associated with perceptual experience, the faster the response time in lexical decision. For individual modalities, visual, auditory, olfactory and interoceptive ratings were significant negative predictors, whereas haptic ratings were a positive predictor (see Table 8). Gustatory ratings were not significant.

**Table 8:** Independent effects of each predictor on lexical decision response time

**Predictor**	**F**	***p***	***R***^**2change**^	**B**_**predictor**_	**RMSE**	**RMSE**_**test**_	**AIC**
Concreteness	156.87	< .001	.004	.095	42.51	42.99	248476
Max. perc. strength	18.16	< .001	.000	– .030	42.51	42.99	248476
Modality exclusivity	23.99	< .001	.001	.024	42.51	42.94	248471
Av. perc. strength	65.53	< .001	.002	– .048	42.47	42.93	248429
Minkowski3	32.47	< .001	.001	– .039	42.50	42.98	248462
Six modalities	20.52	< .001	.003		42.42	42.42	248381
*Visual* *Auditory* *Olfactory* *Gustatory* *Haptics* *Interoceptive*		< .001 < .001 .003 .406 < .001 < .001		– .040 – .020 – .021 – .006 .026 – .034			

All variables were significant independent predictors of lexical decision accuracy. Concreteness and the six individual ratings of each modality led to the biggest improvements over the baseline model. However, concreteness had a negative regression coefficient, indicating higher concreteness lead to lower accuracy. For the six individual variables of perceptual strength, visual, auditory, olfactory and interoceptive ratings were significant positive predictors (see Table 9).

**Table 9:** Independent effects of each predictor on lexical decision accuracy

**Predictor**	**F**	***p***	***R***^**2change**^	**B**_**predictor**_	**RMSE**	**RMSE**_**test**_	**AIC**
Concreteness	49.18	< .001	.001	– .055	.0649	.0645	– 63276
Max. perc. strength	16.74	< .001	.000	.029	.0649	.0645	– 63276
Modality exclusivity	22.52	< .001	.001	– .024	.0649	.0645	– 63281
Av. perc. strength	63.78	< .001	.002	.049	.0648	.0645	– 63323
Minkowski3	33.35	<.001	.001	.041	.0648	.0645	– 63292
Six modalities	17.57	< .001	.003		.0647	.0646	– 63354
*Visual* *Auditory* *Olfactory* *Gustatory* *Haptics* *Interoceptive*		< .001 .009 < .001 .434 .056 < .001		.038 .014 .028 – .006 – .012 .037			

### Study 3b: Does perceptual strength outperform imageability ratings in predicting word processing performance?

### Data and analysis

Analyses were conducted as in Study 3a, but this time ratings of perceptual strength were compared to ratings of imageability. Note that the effects of perceptual strength may differ to Study S1a as imageability ratings were only available for a proportion of the words in the whole data set (5658 compared to 24,039).

### Results and discussion

Only modality exclusivity and the set of six individual modality ratings were significant independent predictors of lexical decision response time. Words that are more multimodal were facilitated in lexical decision. For individual modalities, visual, olfactory and interoceptive ratings were negative predictors of response time, whereas haptic ratings were a positive predictor (see Table 10).

**Table 10:** Independent effects of each predictor on lexical decision response time

**Predictor**	**F**	***p***	***R***^**2change**^	**B**_**predictor**_	**RMSE**	**RMSE**_**test**_	**AIC**
Imageability	1.26	.26	.000	– .016	34.41	34.80	56426
Max. perc. strength	3.17	.07	.000	.026	34.41	34.80	56426
Modality exclusivity	5.21	.02	.001	.025	35.41	34.82	56424
Av. perc. strength	1.41	.24	.000	– .015	35.42	34.85	56428
Minkowski3	1.67	.20	.000	.019	34.79	34.81	56427
Six modalities	18.61	< .001	.013		35.12	34.52	56328
*Visual* *Auditory* *Olfactory* *Gustatory* *Haptics* *Interoceptive*		.21 .07 .06 .08 < .001 < .001		– .020 – .021 – .029 .027 .081 – .085			

For independent effects in accuracy, individual ratings in the six modalities led to the best increase over the model including ratings of imageability, followed by modality exclusivity. Maximum perceptual strength was a negative predictor. Imageability was a positive predictor. Average perceptual strength was not a significant independent predictor (see Table 11). Auditory, olfactory, and interoceptive ratings were positive predictors, whilst haptic ratings were a negative predictor.

**Table 11:** Independent effects of each predictor on lexical decision accuracy

**Predictor**	**F**	***p***	***R***^**2change**^	**B**_**predictor**_	**RMSE**	**RMSE**_**test**_	**AIC**
Imageability	11.08	< .001	.001	.046	.0541	.0524	– 16960
Max. perc. strength	3.51	.06	.000	-.026	.0541	.0524	– 16960
Modality exclusivity	14.62	< .001	.002	-.04	.0540	.0523	– 16971
Av. perc. strength	4.50	.03	.000	.026	.0540	.0524	– 16961
Minkowski3	.34	.56	.000	-.008	.0541	.0524	– 16957
Six modalities	15.86	< .001	.01		.0537	.0522	– 17041
*Visual* *Auditory* *Olfactory* *Gustatory* *Haptics* *Interoceptive*		.97 .01 .01 .06 < .001 < .001		-.000 .028 .040 –.028 –.055 .076			

## General Discussion

We provide new sensory modality ratings for a large set of Dutch words across a range of word classes. The norms are informative with regards to the sensory associations of words in the Dutch language and can be used in linguistic work such as linguistic analyses of the mental lexicon. Additionally, they are a new resource to aid in the selection of stimuli for experimental research (e.g., as in Speed & Majid, 2018) and in the analysis of psycholinguistic variables in mega-studies (Brysbaert et al., 2016). They expand on the existing norms of Speed and Majid (2017) by including a considerably larger number of words, as well as word classes beyond only nouns.

The new norms show some similarities and some differences to previous sets of norms. In our norms and the English norms of Lynott et al. (2020), the largest collection of sensory modality ratings so far, perceptual strength across words followed the same pattern: from vision as strongest, then audition, haptics, interoception, olfaction, and gustation as weakest. There was also a similar pattern in terms of the number of words in each dominant modality. In our norms, the most words were dominant in vision, followed by audition, interoception, gustation, haptics, with the fewest words being dominant in olfaction. This was this same in Lynott et al. (2020) except that haptics had more words than gustation. The new ratings differed somewhat to the previous ratings in Dutch (Speed & Majid, 2017), where haptics had higher strength than audition, and gustatory words were the second most frequent. However, in Speed and Majid (2017) only nouns were rated, and nouns were selected with the goal of having an equal number of words dominant in each modality. The new set of ratings, as well as those of Lynott et al. (2020), include a range of word classes and were not selected with dominant modality in mind. Differences across datasets could also account for differences in modality exclusivity. One way to account for such differences is to compare ratings to a chance level baseline measure specific to each dataset, as done by Winter (2019).

A stable pattern across the sets of norms is the clear visual dominance. Vision consistently has the highest rating and the highest number of words. This is in line with the proposal that vision is easily codable in language (Levinson & Majid, 2014) and is supported by English data from real-world language use where visual words are most frequent across contexts and registers (Winter et al., 2018). This dominance of vision in the lexicon is also reflected in the brain, with visual regions of the brain showing the most extensive functional activation patterns compared to the other sensory modalities (Reilly et al., 2020).

How do the new modality ratings compare with ratings of concreteness and imageability? Concreteness ratings capture ratings in all six modalities to some extent, but with considerable variability in strength: visual and haptic ratings were the strongest predictors of concreteness but the other modalities were more weakly related. This supports the proposal that concreteness ratings do not take into account the full range of sensory experience (Connell & Lynott, 2012). However, in contrast to Connell and Lynott (2012) who found ratings of auditory and gustatory strength negatively predicted concreteness ratings (see also Vergallito et al., 2020), here ratings across all modalities except interoception were positive predictors. Our findings are consistent with a study that collected ratings of only visual and auditory strength for French Canadian nouns (Chedid et al., 2019), where auditory ratings positively correlated with concreteness. We note, however, that auditory and gustatory strength were weak predictors in comparison to the other modalities. This difference with Connell and Lynott (2012) could be due to the large number of adjectives they used, compared to around 18% in the current dataset. In fact, when analyzing only adjectives in our data, auditory strength was also a negative predictor of concreteness (see Supplementary Material S2.2.). For concrete words, results were the same, and for abstract words, the relationship between modality ratings and concreteness was the same except that olfactory ratings were not related to concreteness. In contrast, in Connell and Lynott (2012) vision was a positive predictor of concreteness and audition and olfaction were negative predictors of concreteness for abstract words, and olfaction and vision were positive predictors for concrete words. Again, this pattern is somewhat similar to that observed only for adjectives in our dataset.

As with concreteness, imageability was positively predicted by ratings in all sensory modalities, with vision the strongest, except interoception, which was negative. As suggested by Connell and Lynott (2012), imageability ratings appear to be visually biased. The overall pattern of association however differs to Connell and Lynott (2012) and Vergallito et al. (2020) who found that auditory and gustatory ratings were negative or non-significant predictors of imageability. When looking at only adjectives in the current data, auditory ratings were also a negative predictor, but were not significant (see Supplementary Material S2.3). For high imageability words, we found the same results as Connell and Lynott (2012) with all words, except that gustatory ratings were no longer a significant predictor. For low imageability, gustatory and interoceptive ratings were excluded from the model, and the remaining modalities were positive predictors. This contrasts with Connell and Lynott (2012) where only haptic ratings were a positive predictor and olfactory ratings were a negative predictor.

We also showed that the new sensory norms can predict word processing behavior, and in general were better predictors than concreteness and imageability. Ratings in all six modalities together were the best predictor of lexical decision time and accuracy. Concreteness ratings were a stronger independent predictor of lexical decision response time than the six modalities together, however this was a reverse concreteness effect: higher concreteness ratings led to slower response times. This reverse effect has been observed in other studies (Barber et al., 2013; Brysbaert et al., 2016; Brysbaert et al., 2019; Kousta et al., 2011), although it could reflect a net suppression effect from lexical variables contained in the baseline model. Ratings in the six modalities were the strongest independent predictor of lexical decision accuracy. Across analyses, ratings of the six modalities together are clearly the strongest model of lexical decision response facilitation. This was also the best perceptual predictor in Vergallito et al. (2020; minus interoception ratings), but overall they found imageability to be the strongest predictor of lexical decision performance. This contrasts with Lynott et al., (2020) who found that Minkowski3 was the best predictor of lexical decision. Our findings are also in contrast to Connell and Lynott (2012), who found that maximum perceptual strength was the best predictor. In our analyses, maximum perceptual strength did not consistently outperform the other perceptual measures of modality exclusivity and average perceptual strength. It is important to note here also that we analyzed language processing in terms of performance on a lexical decision task. It is possible that sensory ratings pattern differently with other measures of processing such as naming accuracy and response time (see Connell & Lynott, 2012; Vergallito et al., 2020).

When comparing the sensory ratings across word class, a number of interesting observations can be made (see Supplementary Material). Here, we highlight only a few and hope that future research may explore these differences further. Looking at average perceptual strength across modalities for each word class, it is interesting to note that for adjectives interoception is the second highest rated modality (after vision), whereas for nouns and verbs it is the fourth strongest modality. Since interoception is important for emotional content (Connell et al., 2018), this resonates with the idea that adjectives are less associated with sensory content and more associated with evaluative content than other word classes (Winter, 2019). The importance of interoception for adjectives is also seen in the models assessing the unique contribution of the six-modality model over and above concreteness and imageability. For adjectives, within the six-modality model, interoception is the only significant predictor. This is not the case for nouns and verbs.

Interestingly, number words had the highest modality exclusivity. Number words were primarily associated with vision (average visual rating = 2.25) and weakly associated with the other senses. This could be considered to contradict the proposal that number concepts are amodal and abstract (Nieder, 2016; but see Fischer & Shaki, 2018), however overall the average modality rating for number words is 0.59, which strongly contrasts with nouns (1.08), verbs (1.0), and adjectives (0.91).

Overall there are patterns in the data that differ across word class and across datasets (see Supplementary Material). This highlights on the one hand, the difficulty in making generalizations about the relationship between language and perception, and on the other hand, the potential role of contextual factors in sensory modality ratings (see also Speed et al., 2021). Future work should set out to systematically test the potential influences on sensory modality ratings including linguistic factors (e.g., word class), experiential factors (e.g., individual differences), and procedural factors (e.g., variability in the set of words to rate) in the rating studies.

## Conclusion

We learn words during our multisensory interaction in the world, which leads to sensory information playing a strong role in word meaning. Here we provide a new set of norms that on the one hand supports the proposal that multisensory perceptual information is relevant in word meanings, and on the other hand acts as a resource to further explore the relationship between language and perception. The new set of sensory modality ratings of a large number of Dutch words across word classes will serve as a significant database for researchers assessing semantic representation in the Dutch language. We have demonstrated that the new norms outperform other measures of words’ perceptual associations (concreteness and imageability) in predicting word processing in two different sets of data. We hope that these norms will help in the control and analysis of linguistic stimuli and the selection of experimental items in future scientific work.

## Supplementary Information

ESM 1 (DOCX 60 kb)

## Footnotes

1 The independent effect of concreteness was always tested against a model including baseline variables and maximum perceptual strength, as in Connell and Lynott (2012).

## References

[CR1] Balota DA, Cortese MJ, Sergent-Marshall SD, Spieler DH, Yap MJ (2004). Visual word recognition of single-syllable words. Journal of Experimental Psychology: General.

[CR2] Barber HA, Otten LJ, Kousta S-T, Vigliocco G (2013). Concreteness in word processing: ERP and behavioral effects in a lexical decision task. Brain and Language.

[CR3] Barsalou LW (1999). Perceptions of perceptual symbols. Behavioral and Brain Sciences.

[CR4] Borghi AM, Barca L, Binkofski F, Tummolini L (2018). Varieties of abstract concepts: Development, use and representation in the brain. Philosophical Transactions of the Royal Society B: Biological Sciences.

[CR5] Brysbaert M, Keuleers E, Mandera P (2019). Recognition times for 54 thousand Dutch words: Data from the Dutch Crowdsourcing Project. Psychologica Belgica.

[CR6] Brysbaert M, Stevens M, Mandera P, Keuleers E (2016). The impact of word prevalence on lexical decision times: Evidence from the Dutch Lexicon Project 2. Journal of Experimental Psychology: Human Perception and Performance.

[CR7] Brysbaert M, Warriner AB, Kuperman V (2014). Concreteness ratings for 40 thousand generally known English word lemmas. Behavior Research Methods.

[CR8] Chedid G, Brambati SM, Bedetti C, Rey AE, Wilson MA, Vallet GT (2019). Visual and auditory perceptual strength norms for 3,596 French nouns and their relationship with other psycholinguistic variables. Behavior Research Methods.

[CR9] Chen I-H, Zhao Q, Long Y, Lu Q, Huang C-R (2019). Mandarin Chinese modality exclusivity norms. PLOS ONE.

[CR10] Connell L, Lynott D (2012). Strength of perceptual experience predicts word processing performance better than concreteness or imageability. Cognition.

[CR11] Connell L, Lynott D (2014). I see/hear what you mean: Semantic activation in visual word recognition depends on perceptual attention. Journal of Experimental Psychology: General.

[CR12] Connell L, Lynott D, Banks B (2018). Interoception: The forgotten modality in perceptual grounding of abstract and concrete concepts. Philosophical Transactions of the Royal Society B: Biological Sciences.

[CR13] De Deyne S, Storms G (2008). Word associations: Network and semantic properties. Behavior Research Methods.

[CR14] Filipović Đurđević, D., Popović Stijačić, M., & Karapandžić, J. (2016). A quest for sources of perceptual riches: Several candidates. In *Studies in language and mind* (pp. 187–238). Filozofski fakultet u Novom Sadu.

[CR15] Fischer MH, Shaki S (2018). Number concepts: Abstract and embodied. Philosophical Transactions of the Royal Society B: Biological Sciences.

[CR16] James C (1975). The role of semantic information in lexical decisions. Journal of Experimental Psychology: Human Perception and Performance.

[CR17] Kaschak, M., Zwaan, R. A., Glenberg, A., Morey, R. D., Ibanez, A., Gianelli, C., & Haaf, J. M. (2018). Action-sentence compatibility effect (ACE) pre-registered replication. *Retrieved from osf. io/ynbwu*

[CR18] Kousta S-T, Vigliocco G, Vinson DP, Andrews M, Del Campo E (2011). The representation of abstract words: Why emotion matters. Journal of Experimental Psychology: General.

[CR19] Kroll JF, Merves JS (1986). Lexical access for concrete and abstract words. Journal of Experimental Psychology: Learning, Memory and Cognition.

[CR20] Kuhn, M. (2009). The caret package. *Journal of Statistical Software*, *28*(5).

[CR21] Levinson SC, Majid A (2014). Differential ineffability and the senses. Mind & Language.

[CR22] Lynott D, Connell L (2009). Modality exclusivity norms for 423 object properties. Behavior Research Methods.

[CR23] Lynott D, Connell L (2013). Modality exclusivity norms for 400 nouns: The relationship between perceptual experience and surface word form. Behavior Research Methods.

[CR24] Lynott D, Connell L, Brysbaert M, Brand J, Carney J (2020). The Lancaster sensorimotor norms: Multidimensional measures of perceptual and action strength for 40, 000 English words. Behavior Research Methods.

[CR25] Martin A, Haxby JV, Lalonde FM, Wiggs CL, Ungerleider LG (1995). Discrete cortical regions associated with knowledge of color and knowledge of action. Science.

[CR26] Mazzuca, C., & Borghi, A. M. (2019). Abstract concepts and the activation of mouth-hand effectors. In M. Bolognesi & G. J. Steen (Eds.), *Human Cognitive Processing* (Vol. 65, pp. 43–57). Amsterdam: John Benjamins Publishing Company. 10.1075/hcp.65.03maz

[CR27] Meteyard L, Cuadrado SR, Bahrami B, Vigliocco G (2012). Coming of age: A review of embodiment and the neuroscience of semantics. Cortex.

[CR28] Meteyard L, Zokaei N, Bahrami B, Vigliocco G (2008). Visual motion interferes with lexical decision on motion words. Current Biology.

[CR29] Miklashevsky A (2018). Perceptual experience norms for 506 Russian nouns: Modality rating, apatial localization, manipulability, imageability and other variables. Journal of Psycholinguistic Research.

[CR30] Morucci, P., Bottini, R., & Crepaldi, D. (2019). Augmented modality exclusivity norms for concrete and abstract Italian property words. *Journal of Cognition*, *2*(1): 42, 1-14. 10.5334/joc.88 10.5334/joc.88 PMC6813429 31709385

[CR31] Nieder A (2016). The neuronal code for number. Nature Reviews Neuroscience.

[CR32] Paivio, A. (1986). *Mental Representations: A Dual Coding Approach*. New York: Oxford University Press.

[CR33] Pecher D, Zeelenberg R, Barsalou LW (2003). Verifying different-modality properties for concepts produces switching costs. Psychological Science.

[CR34] Pulvermuller F, Hauk O (2005). Category-specific Conceptual Processing of Color and Form in Left Fronto-temporal Cortex. Cerebral Cortex.

[CR35] R Core Team. (2013). *R: A language and environment for statistical computing*. : R Foundation for Statistical Computing. Retrieved from http://www.R-project.org

[CR36] Reilly J, Flurie M, Peelle JE (2020). The English lexicon mirrors functional brain activation for a sensory hierarchy dominated by vision and audition: Point-counterpoint. Journal of Neurolinguistics.

[CR37] Schwanenflugel PJ, Harnishfeger KK, Stowe RW (1988). Context availability and lexical decisions for abstract and concrete words. Journal of Memory and Language.

[CR38] Speed LJ, Majid A (2017). Dutch modality exclusivity norms: Simulating perceptual modality in space. Behavior Research Methods.

[CR39] Speed LJ, Majid A (2018). An exception to mental simulation: No evidence for embodied odor language. Cognitive Science.

[CR40] Speed, L. J., & Majid, A. (2020). Grounding language in the neglected senses of touch, taste, and smell. *Cognitive Neuropsychology*, *37*(5–6), 363–392. 10.1080/02643294.2019.1623188 10.1080/02643294.2019.1623188 31230566

[CR41] Speed, L., Papies, E. K., & Majid, A. (2021). The role of mental simulation in food attractiveness. 10.31234/osf.io/pdw67

[CR42] Strik Lievers F, Winter B (2018). Sensory language across lexical categories. Lingua.

[CR43] van Dantzig S, Cowell RA, Zeelenberg R, Pecher D (2011). A sharp image or a sharp knife: Norms for the modality-exclusivity of 774 concept-property items. Behavior Research Methods.

[CR44] Van Loon-Vervoorn, A. (1985). *Voorstelbaarheidswaarden van Nederlandse woorden: 4600 substantieven, 1000 verba en 500 adjectieven*. Leiden: Swets & Zeitlinger.

[CR45] Vergallito, A., Petilli, M. A., & Marelli, M. (2020). Perceptual modality norms for 1,121 Italian words: A comparison with concreteness and imageability scores and an analysis of their impact in word processing tasks. *Behavior Research Methods 52, *1599–1616. 10.3758/s13428-019-01337-8 10.3758/s13428-019-01337-8 31950360

[CR46] Verheyen S, De Deyne S, Linsen S, Storms G (2020). Lexicosemantic, affective, and distributional norms for 1,000 Dutch adjectives. Behavior Research Methods.

[CR47] Vincent‐Lamarre, P., Massé, A. B., Lopes, M., Lord, M., Marcotte, O., & Harnad, S. (2016). The latent structure of dictionaries. *Topics in cognitive science, 8*(3), 625–659 10.1111/tops.12211 27424842

[CR48] Winter B (2016). Taste and smell words form an affectively loaded and emotionally flexible part of the English lexicon. Language, Cognition and Neuroscience.

[CR49] Winter, B. (2019). Sensory linguistics: Language, perception, and metaphor. Amsterdam, The Netherlands: Benjamins.

[CR50] Winter B, Perlman M, Majid A (2018). Vision dominates in perceptual language: English sensory vocabulary is optimized for usage. Cognition.

[CR51] Zwaan RA, Stanfield RA, Yaxley RH (2002). Language comprehenders mentally represent the shapes of objects. Psychological Science.

