# Supplementary material for: Dutch sensory modality norms
Source: Behav Res Methods. 2021 Sep 10;54(3):1306–18. doi: 10.3758/s13428-021-01656-9 (PMC9170652; doi:10.3758/s13428-021-01656-9)
Supplement: Supplementary file 1 — (DOCX 60 kb) [file 13428_2021_1656_MOESM1_ESM.docx]

Dutch Sensory Modality Norms: Supplementary Material

Laura J. Speed^1^ & Marc Brybaert^2^

^1^Centre for Language Studies, Radboud University, Nijmegen, Netherlands

^2^Department of Experimental Psychology, Ghent University, Ghent, Belgium

**S1. Correlations between variables**

Table S1. Zero-order correlations between sensory norm ratings, concreteness, and psycholinguistic variables (n = 24,036).

|  | **C** | **V** | **A** | **H** | **G** | **O** | **I** | **ME** | **MMS** | **AMS** | **F** | **P** | **L** | **S** | **Ph** | **OLD** |
| --- | --- | --- | --- | --- | --- | --- | --- | --- | --- | --- | --- | --- | --- | --- | --- | --- |
| **C** | **-** | .70** | -.11** | .62** | .27** | .37** | -.33** | .06** | .69** | .55** | -.03** | .16** | -.25** | -.30** | -.25** | -.10** |
| **V** |  | **-** | -.09** | .56** | .15** | .26** | -.21** | .10** | .82** | .63** | -.02* | .14** | -.15** | -.20** | -.14** | -.04** |
| **A** |  |  | **-** | -.16** | -.16** | -.13** | .22** | -.25** | .15** | .27** | .01 | .05** | .03** | .05** | .02** | -.01 |
| **H** |  |  |  | **-** | .21** | .27** | -.12** | -.30** | .52** | .64** | .00 | .11** | -.18** | -.21** | -.19** | -.14** |
| **G** |  |  |  |  | **-** | .70** | -.04** | -.34** | .25** | .55** | .00 | .02** | -.10** | -.09** | -.10** | -.01* |
| **O** |  |  |  |  |  | **-** | -.06** | -.35** | .330** | .603** | .00 | .04** | -.12** | -.12** | -.11** | -.014* |
| **I** |  |  |  |  |  |  | **-** | -.42** | -.04** | .24** | .01 | .03** | .08** | .07** | .07** | .02* |
| **ME** |  |  |  |  |  |  |  | **-** | .14** | -.50** | -.02** | -.04** | .00 | -.02** | .02* | .05** |
| **MMS** |  |  |  |  |  |  |  |  | **-** | .74** | -.02** | .15** | -.18** | -.22** | -.17** | -.06** |
| **AMS** |  |  |  |  |  |  |  |  |  | **-** | .00 | .14** | -.16** | -.17** | -.16** | -.08** |
| **F** |  |  |  |  |  |  |  |  |  |  | **-** | .04** | -.09** | -.07** | -.09** | -.07** |
| **P** |  |  |  |  |  |  |  |  |  |  |  | **-** | -.04** | -.12** | -.06** | -.13** |
| **L** |  |  |  |  |  |  |  |  |  |  |  |  | **-** | .80** | .93** | .74** |
| **S** |  |  |  |  |  |  |  |  |  |  |  |  |  | **-** | .83** | .59** |
| **Ph** |  |  |  |  |  |  |  |  |  |  |  |  |  |  | **-** | .76** |
| **OLD** |  |  |  |  |  |  |  |  |  |  |  |  |  |  |  | **-** |

* < .05

** <.01

Concreteness (C); Visual (V); Auditory (A); Haptic (H); Gustatory (G); Olfactory (O); Interoception (I); Modality Exclusivity (ME); Maximum Modality Strength (MMS); Average Modality Strength (AMS); Frequency (F); Prevalence (P); Length (L); N Syllables (S); N Phonemes (Ph); OLD

Table S2. Zero-order correlations between sensory norm ratings, imageability and psycholinguistic variables (n = 5658).

|  | **Img** | **V** | **A** | **H** | **G** | **O** | **Int** | **ME** | **MMS** | **AMS** | **F** | **P** | **L** | **S** | **Ph** | **OLD** |
| --- | --- | --- | --- | --- | --- | --- | --- | --- | --- | --- | --- | --- | --- | --- | --- | --- |
| **Img** | **-** | .65^**^ | -.09^**^ | .52^**^ | .18^**^ | .27^**^ | -.25^**^ | -.00 | .62^**^ | .49^**^ | -.04^**^ | .11^**^ | -.12^**^ | -.13^**^ | -.13^**^ | -.05^**^ |
| **V** |  | **-** | -.15^**^ | .54^**^ | .13^**^ | .23^**^ | -.30^**^ | .08^**^ | .79^**^ | .57^**^ | -.08^**^ | .03^*^ | -.11^**^ | -.13^**^ | -.08^**^ | .01 |
| **A** |  |  | **-** | -.22^**^ | -.19^**^ | -.14^**^ | .25^**^ | -.23^**^ | .10^**^ | .23^**^ | -.01 | .06^**^ | .10^**^ | .112^**^ | .072^**^ | .042^**^ |
| **H** |  |  |  | **-** | .20^**^ | .24^**^ | -.22^**^ | -.31^**^ | .50^**^ | .60^**^ | -.04^**^ | 0.02 | -.14^**^ | -.16^**^ | -.14^**^ | -.08^**^ |
| **G** |  |  |  |  | **-** | .70^**^ | -.05^**^ | -.39^**^ | .25^**^ | .59^**^ | -.03^*^ | -0.0 | -.07^**^ | -.04^**^ | -.05^**^ | .02 |
| **O** |  |  |  |  |  | **-** | -.08^**^ | -.41^**^ | .32^**^ | .64^**^ | -.03 | 0.02 | -.08^**^ | -.07^**^ | -.07^**^ | .02 |
| **Int** |  |  |  |  |  |  | **-** | -.39^**^ | -.12^**^ | .16^**^ | .05^**^ | .08^**^ | .09^**^ | .08^**^ | .07^**^ | 0.02 |
| **ME** |  |  |  |  |  |  |  | **-** | .14^**^ | -.56^**^ | -0.01 | -.06^**^ | -.04^**^ | -.06^**^ | -.03^*^ | -.03 |
| **MMS** |  |  |  |  |  |  |  |  | **-** | .69^**^ | -.08^**^ | .04^**^ | -.13^**^ | -.15^**^ | -.11^**^ | -.02 |
| **AMS** |  |  |  |  |  |  |  |  |  | **-** | -.06^**^ | .07^**^ | -.08^**^ | -.08^**^ | -.08^**^ | .00 |
| **F** |  |  |  |  |  |  |  |  |  |  | **-** | .10^**^ | -.12^**^ | -.11^**^ | -.13^**^ | -.13^**^ |
| **P** |  |  |  |  |  |  |  |  |  |  |  | **-** | .06^**^ | .02 | .05^**^ | -.03^*^ |
| **L** |  |  |  |  |  |  |  |  |  |  |  |  | **-** | .84^**^ | .93^**^ | .80^**^ |
| **S** |  |  |  |  |  |  |  |  |  |  |  |  |  | **-** | .85^**^ | .68^**^ |
| **Ph** |  |  |  |  |  |  |  |  |  |  |  |  |  |  | **-** | .81^**^ |
| **OLD** |  |  |  |  |  |  |  |  |  |  |  |  |  |  |  | **-** |

* < .05

** <.01

Imageability (Img); Visual (V); Auditory (A); Haptic (H); Gustatory (G); Olfactory (O); Interoception (Int); Modality Exclusivity (ME); Maximum Modality Strength (MMS); Average Modality Strength (AMS); Frequency (F); Prevalence (P); Length (L); N Syllables (S); N Phonemes (Ph); OLD30

**S2. Analysis of Adjectives**

## S2.1. Summary statistics of adjectives

Adjectives were rated as most strongly associated with vision and weakest in the gustatory modality (see Table S17). As seen in Table S18, the greatest number of words were dominant in the visual modality, and the smallest in the olfactory modality. Table S18 also shows mean modality exclusivity. Words dominant in vision were the most unimodal and words dominant in gustation were the most multimodal. Overall modality exclusivity was 47.5% (*SD* = 14%), which is slightly more multimodal than the full word set.

Table S3. Summary statistics over adjectives per dimension.

| **Modality** | ***M*** | ***SD*** | ***SE*** |
| --- | --- | --- | --- |
| Auditory | 1.07 | 0.80 | 0.012 |
| Gustatory | 0.18 | 0.55 | 0.008 |
| Haptic | 0.64 | 1 | 0.006 |
| Olfactory | 0.18 | 0.46 | 0.007 |
| Visual | 2.20 | 0.98 | 0.015 |
| Interoceptive | 1.21 | 1.00 | 0.016 |

Table S4. Distribution of adjectives over the 6 dominant modalities, with mean ratings and modality exclusivity scores.

|  | **Dominant modality** | | | | | |
| --- | --- | --- | --- | --- | --- | --- |
| **Variable** | **Auditory** | **Gustatory** | **Haptic** | **Olfactory** | **Visual** | **Interoceptive** |
| **Auditory rating** | 2.21 | .38 | .41 | .30 | .85 | 1.25 |
| **Gustatory rating** | .05 | 3.55 | .44 | 1.24 | .13 | .11 |
| **Haptic rating** | .25 | .81 | 3.34 | .60 | .67 | .45 |
| **Olfactory rating** | .05 | 1.52 | .25 | 3.32 | .17 | .12 |
| **Visual rating** | 1.35 | 1.74 | 2.33 | 1.78 | 2.53 | 1.73 |
| **Interoceptive rating** | .96 | .82 | .93 | .89 | .81 | 2.54 |
| **Modality exclusivity** | 46.7% | 40.1% | 43.1% | 41.3% | 50.8% | 41.5% |
| **N** | 425 | 63 | 82 | 19 | 2590 | 878 |

In contrast to the full dataset, not all correlations were significant (see Table S19). Positive correlations were observed between visual and haptic, gustatory, and olfactory ratings, between haptic and gustatory and olfactory ratings, between gustatory and olfactory ratings, and between auditory and interoceptive ratings. Negative correlations were observed between auditory ratings and visual, haptic, gustatory and olfactory ratings, and between interoceptive ratings and visual ratings. Correlations between interoceptive ratings and haptic, gustatory, and olfactory ratings were not significant.

Table S5. Correlations between modalities for adjectives.

|  | **Visual** | **Auditory** | **Haptic** | **Gustatory** | **Olfactory** | **Interoceptive** |
| --- | --- | --- | --- | --- | --- | --- |
| **Visual** | - | -.08** | .44** | .03* | .14** | -.04* |
| **Auditory** |  | - | -.17** | -.10** | -.07** | .39** |
| **Haptic** |  |  | - | .17** | .19** | -.02 |
| **Gustatory** |  |  |  | - | .64** | -.01 |
| **Olfactory** |  |  |  |  | - | .03 |
| **Interoceptive** |  |  |  |  |  | - |

**significant at 0.01 level, *significant at the 0.05 level

## S2.2. Comparing modality ratings with concreteness ratings for adjectives

Data was analysed in the same was as Study 2a in the main manuscript. The best-fitting model contained all 6 modalities *F*(6, 4210) = 672.8, *p* < .001, R^2^ = .49, RMSE = .50. Visual, haptic, and gustatory ratings were significant positive predictors of concreteness, whereas interoceptive and auditory ratings negatively predicted concreteness. Olfactory ratings were not a significant predictor (see Table S20).

We then conducted models separately for concrete (n = 655) and abstract (n=3562) words. For concrete words, olfaction and auditory ratings were excluded from the model. Perceptual strength of the remaining 4 modalities significantly contributed to the regression model of concreteness ratings *F*(4, 650) = 44.44, *p* < .001, R^2^ = .215, RMSE = .372. Visual, haptic, and gustatory ratings positively predicted concreteness, whereas interoceptive ratings negatively predicted concreteness. For abstract words, ratings of olfaction were excluded from the model. Perceptual strength in the remaining 54 modalities significantly contributed to the regression model of concreteness ratings *F*(5, 3556) = 250.8, *p* < .001, R^2^ = .261, RMSE = .347. Visual, haptic, and gustatory ratings significantly positively predicted concreteness, whereas auditory ratings negatively predicted concreteness (although the *p*-value failed to meet the conventional significance level; *p* = .052). Interoception was not a significant predictor in the model.

Table S6. Standardized regression coefficients for each dimension as predictors of concreteness of adjectives. All coefficients significant at p < .001.

| **Rating** | **Visual** | **Auditory** | **Haptic** | **Gustatory** | **Olfactory** | **Interoceptive** |
| --- | --- | --- | --- | --- | --- | --- |
| Concreteness | .46 | -.09 | .28 | .14 | .02 | -.12 |
| Concreteness: Concrete | .35 | - | .14 | .17 | - | -.20 |
| Concreteness: Abstract | .40 | -.03 | .18 | .06 | - | -.02 |

## S2.3. Comparing modality ratings with imageability ratings for adjectives

The same analyses were conducted to compare modality ratings with imageability ratings. The best-fitting model contained all 4 modalities *F*(4, 866) = 184.1, *p* < .001, R^2^ = .460, RMSE = .805, with audition and olfaction removed. Vision, touch, and interoception were significant positive predictors of imageability (see Table S21).

We next conducted models separately for high and low imageability. For high imageability, ratings of visual, auditory, and haptic strength were included in the final model, *F*(3, 618) = 122.2, *p* < .001, R^2^ = .372, RMSE = .608. Visual and haptic ratings positively predicted imageability, and auditory ratings negatively predicted imageability. For low imageability, ratings of visual, olfactory and interoceptive strength were included in the final model, *F*(3, 245) = 11.46, *p* < .001, R^2^ = .123, RMSE = .434, but only visual and interoceptive ratings were positive predictors of imageability.

Table S7. Standardized regression coefficients for each dimension as predictors of imageability of adjectives. ** = significant at *p* < .001, * significant at *p* < .05

| **Rating** | **Visual** | **Auditory** | **Haptic** | **Gustatory** | **Olfactory** | **Interoceptive** |
| --- | --- | --- | --- | --- | --- | --- |
| Imageability | 0.622** | - | 0.123** | 0.068* | - | 0.079** |
| Imageability: High | 0.569** | -0.082** | 0.066** | - | - | - |
| Imageability: Low | 0.257** | - | - | - | 0.082 | 0.132* |

# S3. Analysis of Nouns

## S3.1. Summary statistics of nouns

Nouns were rated as primarily experienced through vision, and least in the gustatory modality (see Table S30). Mean haptic ratings are slightly higher than for the full word set, but in general the pattern is the same. As seen in Table S31, the greatest number of words were dominant in the visual modality, and the smallest in the olfactory modality. Table S31 also shows mean modality exclusivity. Words dominant in vision were the most unimodal and words dominant in gustation were the most multimodal. Overall modality exclusivity was 50.9% (SD = 14%), which is slightly more unimodal than the full word set.

Table S8. Summary statistics per dimension for nouns.

| **Modality** | ***M*** | ***SD*** | ***SE*** |
| --- | --- | --- | --- |
| Auditory | 1.11 | 1.03 | .009 |
| Gustatory | 0.31 | .91 | .008 |
| Haptic | 1.12 | 1.04 | .009 |
| Olfactory | 0.39 | .78 | .007 |
| Visual | 2.89 | 1.04 | .009 |
| Interoceptive | 0.63 | .006 | .765 |

Table S31. Distribution of nouns over the 6 dominant modalities, with mean ratings and modality exclusivity scores.

|  | **Dominant modality** | | | | | |
| --- | --- | --- | --- | --- | --- | --- |
| **Variable** | **Auditory** | **Gustatory** | **Haptic** | **Olfactory** | **Visual** | **Interoceptive** |
| **Auditory rating** | 3.00 | .21 | .73 | .43 | .94 | 1.10 |
| **Gustatory rating** | .04 | 4.17 | .17 | .96 | .16 | .13 |
| **Haptic rating** | .44 | 1.82 | 3.35 | 1.05 | 1.16 | .50 |
| **Olfactory rating** | .07 | 2.58 | .32 | 3.60 | .31 | .13 |
| **Visual rating** | 1.77 | 3.32 | 2.79 | 2.26 | 3.11 | 1.59 |
| **Interoceptive rating** | .75 | .39 | .92 | .65 | .47 | 2.59 |
| **Modality exclusivity** | 50.9% | 32.9% | 40.5% | 41.6% | 53.0% | 43.8% |
| **N** | 1288 | 272 | 282 | 120 | 11112 | 799 |

All modalities were significantly correlated (see Table S32). Positive correlations were observed between visual and haptic, gustatory, and olfactory ratings, between haptic and gustatory and olfactory ratings, between gustatory and olfactory ratings, and between auditory and interoceptive ratings. Negative correlations were observed between auditory ratings and visual, haptic, gustatory and olfactory ratings, and between interoceptive ratings and visual, haptic, gustatory, and olfactory ratings.

Table S9. Correlations between modalities. All correlations p < .001.

|  | **Visual** | **Auditory** | **Haptic** | **Gustatory** | **Olfactory** | **Interoceptive** |
| --- | --- | --- | --- | --- | --- | --- |
| **Visual** | - | -.12** | .57** | .16** | .25** | -.27** |
| **Auditory** |  | - | -.20** | -.20** | -.16** | .19** |
| **Haptic** |  |  | - | .23** | .28** | -.12** |
| **Gustatory** |  |  |  | - | .71** | -.05** |
| **Olfactory** |  |  |  |  | - | -.08** |
| **Interoceptive** |  |  |  |  |  | - |

## S3.2. Comparing modality ratings with *concreteness* ratings for nouns

Data was analysed in the same was as Study 2a in the main manuscript. Perceptual strength on all six modalities contributed to the regression model of concreteness ratings *F*(6, 14391) = 3955, *p* < .001, R^2^ = .6225, RMSE = .6318. Visual, auditory, haptic, gustatory and olfactory ratings were significant positive predictors of concreteness, whereas interoceptive ratings negatively predicted ratings of concreteness.

We then conducted models separately for concrete and abstract nouns. For concrete nouns, auditory ratings were excluded from the final model. Perceptual strength on remaining five modalities contributed to the regression model of concreteness ratings *F*(5, 8726) = 1284, *p* < .001, R^2^ = .424, RMSE = .4452. Visual, haptic, gustatory and olfactory ratings were significant positive predictors of concreteness, whereas interoceptive ratings negatively predicted ratings of concreteness. For abstract nouns, olfactory ratings were excluded. Perceptual strength remaining five modalities contributed to the regression model of concreteness ratings *F*(5, 5659) = 308, *p* < .001, R^2^ = .214, RMSE = .3876. Visual, haptic, and gustatory ratings were significant positive predictors of concreteness, whereas interoceptive ratings negatively predicted ratings of concreteness. See Table S33 for standardized regression coefficients.

Table S10. Standardized regression coefficients for each dimensions as predictors of concreteness of nouns. All coefficients significant at p < .001.

| **Rating** | **Visual** | **Auditory** | **Haptic** | **Gustatory** | **Olfactory** | **Interoceptive** |
| --- | --- | --- | --- | --- | --- | --- |
| Concreteness | 0.40 | 0.01 | 0.35 | 0.03 | 0.14 | -0.18 |
| Concreteness: (Concrete) | 0.25 | - | 0.41 | 0.08 | 0.15 | -0.12 |
| Concreteness: (Abstract) | 0.32 | 0.03 | 0.14 | 0.05 | - | -0.23 |

## S3.3. Comparing modality ratings with *imageability* ratings for nouns

The same analyses were conducted to compare modality ratings with imageability ratings. Perceptual strength in vision, olfaction, haptics and interoception contributed to the regression model of imageability ratings *F*(4, 3831) = 1071, *p* < .001, R^2^ = .528, RMSE = .8845. Ratings in the visual, haptic and olfactory modality were significant positive predictors of imageability, whereas ratings in interoception were a negative predictor.

The next step was to conduct the models separately for high and low imageability.

For high imageability nouns, auditory and gustatory ratings were excluded from the final model. Perceptual strength in remaining four modalities contributed to the regression model of imageability ratings, *F*(4, 3024) = 391, *p* < .001, R^2^ = .341, RMSE = .6484. Ratings in the visual, haptic, and olfactory modalities positively predicted imageability, whereas ratings in the interoceptive modality negatively predicted imageability. For low imageability nouns, interoceptive ratings were excluded from the final model. Perceptual strength remaining five modalities contributed to the regression model of imageability ratings, *F*(5, 801) = 14.33, *p* < .001, R^2^ = .082, RMSE = .5436, all of which were significant positive predictors except for gustation. See Table S34 for standardized regression coefficients.

Table S11. Standardized regression coefficients for each dimensions as predictors of imageability of nouns.

| **Rating** | **Visual** | **Auditory** | **Haptic** | **Gustatory** | **Olfactory** | **Interoceptive** |
| --- | --- | --- | --- | --- | --- | --- |
| Imageability | .489* | - | .213* | - | .126* | -.124* |
| Imageability: High | .369* | - | .208* | - | .144* | -.125* |
| Imageability: Low | .220* | .105* | .083* | -.08 | .12* | - |

# S4. Analysis of Verbs

## S4.1. Summary statistics of verbs

Verbs were rated as mostly strongly associated with vision and least with gustation (see Table S43), the same pattern as observed in the full set of words. As seen in Table S44, the greatest number of words were dominant in the visual modality, and the smallest in the olfactory modality. Table S44 also shows mean modality exclusivity. Words dominant in audition were the most unimodal and words dominant in gustation were the most multimodal. Overall modality exclusivity was 47.6% (*SD* = 12.9%), which is slightly more multimodal than the whole set of words.

Table S12. Summary statistics per dimension for verbs.

| **Modality** | ***M*** | ***SD*** | ***SE*** |
| --- | --- | --- | --- |
| Auditory | 1.30 | 1.05 | .015 |
| Gustatory | .13 | .43 | .006 |
| Haptic | 1.11 | .97 | .014 |
| Olfactory | .18 | .49 | .007 |
| Visual | 2.46 | .92 | .013 |
| Interoceptive | .79 | .78 | .011 |

Table S13. Distribution of verbs over the 6 dominant modalities, with mean ratings and modality exclusivity scores.

|  | **Dominant modality** | | | | | |
| --- | --- | --- | --- | --- | --- | --- |
| **Variable** | **Auditory** | **Gustatory** | **Haptic** | **Olfactory** | **Visual** | **Interoceptive** |
| **Auditory rating** | 2.96 | .68 | .94 | .79 | .97 | 1.22 |
| **Gustatory rating** | .05 | 3.30 | .15 | 1.45 | .11 | .16 |
| **Haptic rating** | .48 | 1.27 | 3.15 | 1.16 | 1.16 | .57 |
| **Olfactory rating** | .07 | 1.59 | .18 | 3.78 | .18 | .14 |
| **Visual rating** | 1.66 | 2.29 | 2.49 | 2.29 | 2.72 | 1.71 |
| **Interoceptive rating** | .84 | .89 | .89 | .70 | .62 | 2.49 |
| **Modality exclusivity** | 50.0% | 30.2% | 41.2% | 37.7% | 48.9% | 39.8% |
| **N** | 739 | 26 | 222 | 22 | 3326 | 300 |

Table S14. Correlations between modalities for verbs. All correlations p < .001.

|  | **Visual** | **Auditory** | **Haptic** | **Gustatory** | **Olfactory** | **Interoceptive** |
| --- | --- | --- | --- | --- | --- | --- |
| **Visual** | - | -.07** | .51** | .11** | .19** | -.07** |
| **Auditory** |  | - | -.11** | -.03 | -.02 | .20** |
| **Haptic** |  |  | - | .11** | .16** | -.08** |
| **Gustatory** |  |  |  | - | .59** | .07** |
| **Olfactory** |  |  |  |  | - | .03** |
| **Interoceptive** |  |  |  |  |  | - |

## S4.2. Comparing modality ratings with *concreteness* ratings for verbs

Data was analyzed in the same was as Study 2a in the main manuscript. Perceptual strength on all six modalities contributed to the regression model of concreteness ratings *F*(6, 4740) = 767.3, *p* < .001, R^2^ = .4927, RMSE = .5089. Visual, auditory, haptic, gustatory and olfactory ratings were significant positive predictors of concreteness, whereas interoceptive ratings negatively predicted ratings of concreteness.

We then conducted models separately for concrete and abstract words. For concrete words, ratings of taste were excluded from the final model. Perceptual strength on the remaining five modalities contributed to the regression model of concreteness ratings *F*(5, 2238) = 107.4, *p* < .001, R^2^ = .1918, RMSE = .3622. Visual, haptic, auditory olfactory ratings were significant positive predictors of concreteness. Interoceptive ratings did not significantly predict ratings of concreteness. For abstract words, ratings in all six modalities contributed to the regression model of concreteness ratings *F*(6, 2496) = 122.3, *p* < .001, R^2^ = .2254, RMSE = .3105. Visual, haptic, and auditory ratings were significant positive predictors of concreteness, whereas interoceptive ratings negatively predicted ratings of concreteness. Smell and taste were not significant predictors. See Table S45 for standardized regression coefficients.

Table S15. Standardized regression coefficients for each dimensions as predictors of concreteness of verbs.

| **Rating** | **Visual** | **Auditory** | **Haptic** | **Gustatory** | **Olfactory** | **Interoceptive** |
| --- | --- | --- | --- | --- | --- | --- |
| Concreteness | 0.43* | 0.16* | 0.31* | 0.03* | 0.10* | -0.15* |
| Concreteness: (Concrete) | 0.25* | 0.20* | 0.24* | - | 0.15* | -0.04 |
| Concreteness: (Abstract) | 0.311* | 0.09* | 0.22* | -0.01 | 0.04 | -0.16* |

## S4.3. Comparing modality ratings with *imageability* ratings of for verbs

The same analyses were conducted to compare modality ratings with imageability ratings. Perceptual strength on all six modalities contributed to the regression model of concreteness ratings *F*(6, 944) = 112.4, *p* < .001, R^2^ = .4168, RMSE = .8648. Visual, auditory, gustatory and olfactory ratings were significant positive predictors of concreteness, whereas interoceptive ratings negatively predicted ratings of concreteness. Gustatory ratings were not a significant predictor. The next step was to conduct the models separately for high and low imageability. For high imageability verbs, interoceptive ratings were excluded from the final model. Perceptual strength remaining five modalities contributed to the regression model of imageability ratings, *F*(5, 783) = 57.86, *p* < .001, R^2^ = .270, RMSE = .6590, all of which were positive predictors, except gustatory ratings were not a significant predictor. For low imageability verbs, the final model included only auditory and visual ratings, *F*(2, 159) = 3.46 *p* = .034, R^2^ = .042, RMSE = .4985. See Table S46 for standardized regression coefficients.

Table S16. Standardized regression coefficients for each dimensions as predictors of imageability of verbs.

| **Rating** | **Visual** | **Auditory** | **Haptic** | **Gustatory** | **Olfactory** | **Interoceptive** |
| --- | --- | --- | --- | --- | --- | --- |
| Imageability | .476** | .210** | .223** | .034 | .086* | -.030 |
| Imageability: High | .391** | .150** | .185** | .051 | .107* | - |
| Imageability: Low | .148 | .161* | - | - | - | - |
